# The Eukaryotic Flagellum Makes the Day: Novel and Unforeseen Roles Uncovered After Post-Genomics and Proteomics Data

**DOI:** 10.2174/138920312803582951

**Published:** 2012-09

**Authors:** Michely C Diniz, Ana Carolina L Pacheco, Kaio M Farias, Diana M de Oliveira

**Affiliations:** 1 Programa de Pós-Graduação em Biotecnologia-RENORBIO-Rede Nordeste de Biotecnologia, Universidade Estadual do Ceará-UECE, Av. Paranjana, 1700, Campus do Itaperi, Fortaleza, CE 60740-000 Brasil; 2 Colegiado de Ciências Biológicas, Universidade Federal do Vale do São Francisco-UNIVASF, Campi Ciências Agrárias-Rodovia BR 407, Projeto de Irrigação-Nilo Coelho-S/N, Petrolina, PE, 56300-000 Brasil; 3 Unidade Acadêmica de Ciências Biológicas e Nutrição, Universidade Federal do Piauí-UFPI, Picos, PI, Brasil; 4 Curso de Mestrado Acadêmico em Nutrição e Saúde –CMANS, Centro de Ciências da Saúde, Universidade Estadual do Ceará-UECE, Av. Paranjana, 1700, Campus do Itaperi, Fortaleza, CE 60740-000 Brasil

**Keywords:** Eukaryotic flagella, cilia, flagellar proteins, flagellar proteome, axoneme, intraflagellar transport, motility, pathogenic flagellates.

## Abstract

This review will summarize and discuss the current biological understanding of the motile eukaryotic flagellum, as posed out by recent advances enabled by post-genomics and proteomics approaches. The organelle, which is crucial for motility, survival, differentiation, reproduction, division and feeding, among other activities, of many eukaryotes, is a great example of a natural nanomachine assembled mostly by proteins (around 350-650 of them) that have been conserved throughout eukaryotic evolution. Flagellar proteins are discussed in terms of their arrangement on to the axoneme, the canonical “9+2” microtubule pattern, and also motor and sensorial elements that have been detected by recent proteomic analyses in organisms such as *Chlamydomonas reinhardtii*, sea urchin, and trypanosomatids. Such findings can be remarkably matched up to important discoveries in vertebrate and mammalian types as diverse as sperm cells, ciliated kidney epithelia, respiratory and oviductal cilia, and neuro-epithelia, among others. Here we will focus on some exciting work regarding eukaryotic flagellar proteins, particularly using the flagellar proteome of *C. reinhardtii* as a reference map for exploring motility in function, dysfunction and pathogenic flagellates. The reference map for the eukaryotic flagellar proteome consists of 652 proteins that include known structural and intraflagellar transport (IFT) proteins, less well-characterized signal transduction proteins and flagellar associated proteins (FAPs), besides almost two hundred unannotated conserved proteins, which lately have been the subject of intense investigation and of our present examination.

## 1 THE EUKARYOTIC FLAGELLUM

From the early days of first reports on flagellum [1-8], there has been quite a long way of improvements towards current knowledge [9-18] and our general understanding of how this amazing organelle manages its diversity of biological functions and roles. It would be impossible to introduce the subject of eukaryotic^1^ flagellar proteins without mentioning the work of important pioneers such as Joel Rosembaum, Ian Gibbons and Gianni Piperno, among others, who have continuously contributed to the field of flagellum/cilium research in a long, careful, elegant and meticulous series of studies that exploited *Chlamydomonas reinhardtii* flagella as a model system for studying the biogenesis of subcellular organelles, culminating with the astounding discovery of the intraflagelar transport mechanism (IFT) for explaining the intense transit of motor proteins along the axoneme [9, 19; for review see 16, 20]. Adding up to these brilliant views on the flagellum, we will bring up the post-genomics view comprising the “sequence-structure-function” triad [21-25] that has been employed here to address state-of-the-art flagellar proteomics. 

Proteomics, as the understanding of the huge number and range of proteins that are expressed by a cell type or organelle under any given set of conditions is evolving rapidly to cope with a range of heterogeneous source of protein sequences, information, structures, functions and their overall interpretations and derived inferences. Protein databases are growing at a hasty rate and, in recent years, the necessity of reliable bioinformatics methods and resources for studying protein functions and interactions is simply overwhelming. Organelle proteomics [26-28], such as the flagellum [13, 29, 30] and sub-proteomes as the recently published flagellar phosphoproteome [18] and others [31], has been one of the most suitable approaches used to develop a better understanding of the complete proteome profiles of a given organism. The power of such proteome approaches lies in the identification of novel components and modifications of the given tissue, organelle or sub-proteome (in this case the flagellum and a few related sub-proteomes) that have not been discovered before. Information is usually gained at a large scale and is very valuable to further understand biological processes of the given compartment [31].

Our review interest is mainly about flagellar proteins expressed in one or more compartments of the eukaryotic cilium/flagellum (the terms can be used as equivalent), since these proteins contribute to our understanding of how rather small proteomes (not exceeding from 400-600 proteins) [13, 25, 29, 30] lead to flagella being so different from one to another (number, size, remodeling, paraflagellar structures, and other features) in a wide range of organisms. As long or short projections, thread-like or antenna-like organelles that are surrounded by a specialized extension of the cell membrane, flagella Fig. (**1a**) and (**1b**)) comprise complex and dynamic functions associated to genes encoding isoforms of ubiquitous and unique proteins within eukaryotes [reviewed by 15, 32]. Research conducted by several groups in model organisms, such as *C. reinhardtii*, sea urchin, trypanosomatids and mammalian sperm, among many others [16, 30, 33-42], has impressively increased our knowledge about this intriguing organelle that is a key part of the motile, dividing and invasive machinery of many eukaryotes. 

Eukaryotic cilia and flagella are microtubule-based cellular extensions, which play critical roles in cell motility, development and sensory perception. They interact with the environment through signal transduction and gene expression networks [15, 43, 44-46], while their important and recently uncovered roles played in several human diseases and physiological conditions create an urgent need to identify genes involved in ciliary assembly and function [28, 39, 45, 47-49]. As complex organelles, comprising a few hundreds of distinct polypeptides and proteins assembled onto a framework of microtubules [33, 34], eukaryotic flagella and cilia have one defining feature: the 9+2 axoneme characterized by nine outer doublet microtubules and two central pair singlet microtubules Fig. (**2**). The nine outer doublets slide relative to one another; dynein arms generate the force for this sliding; radial spokes connect outer doublets with central singlets [33, 34]. The transient association of dynein arms attached to one doublet with an adjacent doublet results in microtubule sliding, while the constraints on doublet movement convert sliding into bending force. The resultant flagellar beating can be crucial for motility, survival, reproduction or feeding of many eukaryotes. It is believed that the last common ancestor of extant eukaryotes had flagella (possibly a pair) [50], and many of the proteins assembled on to the axoneme have been conserved throughout eukaryotic evolution [37, 47, 51]. 

In general, cilia are classified as motile (9+2) or nonmotile (9+0), the latter, also known as primary cilia, are present on most cells in the mammalian body [52]. Although first described as early as 1898 and long considered a vestigial organelle of little functional importance, the primary cilium has become one of the hottest research topics in modern cell biology and physiology due to defects in its assembly or function that have been tightly coupled to many developmental defects, diseases and disorders. In normal tissues, the primary cilium coordinates a series of signal transduction pathways, including Hedgehog (hh) and Sonic Hedgehog (Shh), Wnt, the platelet-derived growth factor receptor (PDGFR) alpha, and integrin signaling. In the kidney, the primary cilium may function as a mechano-, chemo- and osmosensing unit that probes the extracellular environment and transmits signals to the cell via, e.g., polycystins, which depend on ciliary localization for appropriate function. Indeed, hypomorphic mutations in the mouse ift88 (previously called Tg737) gene, which encodes a ciliogenic IFT protein [53], result in malformation of primary cilia, and in the collecting ducts of kidney tubules this is accompanied by development of autosomal recessive polycystic kidney disease (PKD). While PKD was one of the first diseases to be linked to dysfunctional primary cilia, defects in this organelle have subsequently been associated with many other phenotypes, including cancer, obesity, diabetes as well as a number of developmental defects. Collectively, these disorders of the cilium are now referred to as the ciliopathies [52, 54].

Altogether, cilia and flagella (motile, sensory or primary), comprise one of the most highly conserved structures in eukaryotes and an exciting field of research that has provided amazing results with great impact in several areas of biomedical sciences, including unforeseen roles and sites of action for cilia and flagellar proteins [28, 39, 41, 55-58). Therefore, flagellar genes and proteins, identified and analyzed by multiple post-genomics and proteomics approaches, are the most promising candidates for current and future studies to test whether the encoded and/or expressed proteins are sufficient and necessary for flagella/cilia function, dysfunction and overall activity in studied organisms, particularly using the flagellar proteome of *C. reinhardtii* [13] as a reference map (Table **1**).

In this review we will focus on some of the most exciting findings, obtained through proteomics analyses, regarding flagellar proteins on eukaryotic motility (Table **2**) and derived activities, on the sense that they have attracted tremendous attention, in recent years, due to unanticipated crucial roles in coordinating a number of physiologically and developmentally important pathways related to flagellar motility in function and dysfunction and also in pathogenic flagellates. 

## 2 CONSERVED TO DIVERGE IN FLAGELLAR ASSEMBLY AND DISASSEMBLY

Eukaryotic flagella are thought to have played a key role in the development of multicellularity by performing tasks, mostly dependent on the microtubule-based construction, through the coordinated action of groups of flagella (from a single one to several pairs), which display various types of spatio-temporal organization [59, 60]. Differences in number, size, differentiated states and remodeling fashions are remarkable in eukaryotic flagella. Just to mention an outstanding comparative example coming from three protozoan parasites, e.g. *Plasmodium*, *Giardia lamblia* and *Trichomonas*: *Plasmodium* possesses two long flagella that are free from association with the gamete body and a third shorter flagellum (or rudimentary) that is attached to surface of gamete at its anterior end [reviewed by 61, 62]; *Giardialamblia* possesses four pairs of flagella and *Trichomonas* four free flagella and a fifth recurrent one [63]. In many eukaryotic species, this organization of flagella will be present in assorted and distinctive forms, one of which have been mostly studied and became a model: the biflagellate green alga *C. reinhardtii*. 

*Chlamydomonas* is ideal for an integrated view of flagellum function because its genetics is similar to yeast (relative adaptability and quick generation time), but, unlike yeast, *Chlamydomonas* has two flagella that are virtually identical to human cilia [32]. Since sufficient biological material is easily available to efficiently establish biochemical purification procedures of sub-cellular fractions, this green alga is also an excellent model for proteomics research as well. 

Most of the previously identified human ciliary disease genes have orthologs in *Chlamydomonas* that have been shown to be involved in flagellar assembly [64]. *C. reinhardtii* provides an excellent model system for investigating flagellar gene expression network responses, since its two flagella act as environmental sensors for the cell. Changing the cell's environment in various ways causes changes in flagellar morphology. For example, experimental acidification of the medium (acid shock) induces flagellar excision [65] followed by regrowth or assembly of new flagella within 2 hr [66, 67]. It has been shown that stimulation of *Chlamydomonas* by treatment with IBMX (3-isobutyl-1-methylxanthine), for example, induces flagellar resorption or shortening of the flagella (referred to as disassembly) [67, 68]. Resorption, in turn, is known to be reversible with the additional complication that the resorbed flagellar components can be re-utilized to assemble flagella in the absence of protein synthesis. Synthesis of flagellar proteins is stimulated after cells are chemically induced to resorb their flagella [66-68]. This reinforces the complexity of deflagellation and reflagellation (also called flagellar disassembly and assembly) as multifaceted events [68-70] with conflicting evidences on the synthesis of different flagellar proteins required to regenerate full-length flagella after deflagellation.* Chlamydomonas* cells can be induced to shed their flagella via katanin-mediated severing, after which the flagella immediately begin to regenerate. During this process, it has been shown that many known flagellar proteins are transcriptionally induced, with most transcripts reaching maximum accumulation between 30-45 minutes [69] after deflagellation [70, 32]. In contrast, genes encoding components of other organelles do not show this induction in response to deflagellation [32, 71]. 

Coupled with the availability of the *Chlamydomonas* genome (http://genome.jgi-psf.org/Chlre4) [72] and 232,208 expressed sequence tags (ESTs) processed from various public sources by Chlamy EST-Terminus [73], these observations propose a systematic strategy for flagellar/ciliary gene identification, which provides further assistance to improved proteomic approaches [32]. 

The flagellar proteome of *C. reinhardtii* has been used as a reference map for proteomics research on cilia and flagella since its publication [13], with over 400 citations, and we here emphasize its extreme importance for the field. The reference map consists of an estimated total of 652 proteins (identified by Pazour and colleagues through mass spectrometry (MS) and depicted here in a simplified view on Table **1**); 360 proteins identified by five or more peptides (most likely to be true flagellar proteins) and 292 identified by two to four peptides (likely to be candidate flagellar proteins that need to be confirmed by further analysis). We must reinforce that 122 are known flagellar proteins (distributed in thirteen groups of structural and transport proteins), as well as approximately 200 less well-characterized signal transduction proteins and flagellar associated proteins (FAPs), besides more than a hundred conserved proteins unannotated [13, 30]. 

Many investigations have demonstrated coordination between flagellar gene expression and flagellar assembly and disassemby. During the course of flagellar assembly, for example, genes encoding alpha- and β-tubulin are transiently upregulated and return to prestimulation levels as the regenerating flagella reach full length [68, 74-77]. Recent genomic [32] and proteomic [13] studies have profiled similar expression patterns for large numbers of additional genes during flagellar assembly, using the reference map for the flagellar proteome of *C. reinhardtii* [13].

### 2.1 The Intraflagellar Transport (IFT) Genes and Proteins

Studies across eukaryotic systems indicate that flagella are constructed (assembled) and maintained through the highly conserved process of IFT [9, 19, 20]. Well characterized in *Chlamydomonas*, IFT is a rapid movement of particles along the axonemal microtubules of cilia and flagella, being a specialized bidirectional transport process mediated by the ancestral and conserved IFT complex. The import and export of proteins appear to be largely mediated by IFT particles that move along the axonemal doublet microtubules just beneath the flagellar membrane [12, 19, 78] and are associated either with kinesin or with dynein motor proteins, recycling kinesin and discarding axoneme proteins back to the cytosol [79]. It has been reported that IFT is not only required for building cilia/flagella, but also directly involved in sensory signal transduction in *Chlamydomonas* [80] and secretory functions [81].

The IFT system consists of anterograde (from the cell body to the ciliary tip) and retrograde (from the ciliary tip to the cell body) motor complexes associated with raft-like large protein complexes called IFT particles. Genetic and biochemical analyses in *C. reinhardtii* and *Caenorhabditis elegans* have identified IFT motor subunits as well as many of the IFT particle components [reviewed in 12, 16, 82, 83]. The main function of IFT is likely to be the delivery of axonemal substructures from the basal body region to the distal end of the flagellum, where the axoneme assembles [84-86]. The particles that are transported by IFT are composed of several protein subunits [87, 88]. Exact functions of the individual subunits are not known, but the proteins are well conserved between green algae, nematodes, and vertebrates [20, 88]. In *Chlamydomonas*, IFT particles comprise two large complexes: complex A is composed of seven subunits (IFT42, IFT121, IFT122A/B, IFT139, IFT140, and IFT144/148); complex B is composed of fifteen subunits (IFT20, IFT22, IFT25, IFT27, IFT46, IFT52, IFT54, IFT57, IFT70, IFT74/72, IFT80, IFT81, IFT88, and IFT172) [42, 88]. Therefore, IFT complex is now estimated to be composed of at least 22 different polypeptides, including the recently reported IFT25 [42, 89-91], which is homologous to the human heat shock protein family B (small) member 11, as well as IFT70 [91] and the recently described subcomplex IFT144/140/122 [92].

Even though significant strides have been made in dissecting the mechanisms of IFT, it remains a poorly understood process, including its structure and architecture [92]. For instance, the full complement of its components is not yet known and the organization, regulation, and specific functions and molecular structure of the IFT machinery are incompletely understood [92, 93, 42]. Exciting recent advances have linked IFT not only with the delivery of ciliary components required for the assembly, maintenance, and length control of motile and sensory cilia but also for carrying cilium-based signals that control cell function, gene expression, cell division, animal development, and the onset of some human diseases [94-96]. Given the important biological functions of IFT, the development of a precise understanding of how IFT particles and their associated proteins are moved, as cargo along the flagellum/cilium [16], will continue to be a priority.

There has been an increase in the identification of IFT homologs in the last years, although only the homologs of the classical components (IFT88, -57, 52- and -20) had been *in vitro* identified in all studied eukaryotic flagellate/ciliate models and also in human cells [as reviewed by 12]. There are reports of homologs to members of the IFT complex proteins in several organisms, including trypanosomatids such as *Trypanosoma* [40, 97-100] and *Leishmania*, the latter that has been our own focus upon the eukaryotic flagellum [101-103]. The first work to provide the actual demonstration of IFT in *Trypanosoma brucei* [100] also revealed the activity of this process in both old (in maintenance) and new flagellum (in construction) in the same cell. When the new flagellum is assembled, incorporation of new subunits takes place at its distal tip, whereas only a small amount of material is turned over in the old flagellum [104]. That report demonstrated the restricted location of IFT particles to two sets of specific outer doublets (3–4 and 7–8) in *T. brucei*. It has been argued [40] that such restricted location could be partially explained by physical constraints resulting from the presence of the extra-axonemal PFR [105]. IFT proteins are abundant at the base of the flagellum, where they localize to the apical region of the basal body [40, 106].

Although the coordination of structure and gene expression is well characterized for flagellar assembly, the knowledge about gene regulation during disassembly had been largely limited to a known decrease in expression of alpha- and β-tubulin mRNA levels [68, 71, 74]. Recently, important roles played by IFT in flagellar assembly and disassembly have called attention [44], such as the transport of flagellar components along the length of the axoneme [83] and putative actions to regulate flagellar length [107, 108] and assembly highlighted in IFT52 roles (BLD1/osm-6) [109]. 

The first IFT proteins to have a crystal structure deposited at PDB were the *Chlamydomonas* IFT25 and IFT27 [PDB 2YC2 and 2YC4] [110], which comprise a complex (IFT25/IFT27) that interact via a conserved interface seen on Fig. (**3**). Recent results on IFT subunits structure and function [92, 110] will certainly provide big steps towards a better understanding of the IFT complex. 

### 2.2 Flagellar Associated Proteins (FAPs)

The flagellar proteome [13] contains at least 60 less well-characterized flagellar associated proteins (FAPs), whose importance will eventually increase as functions are described for these particular FAPs not yet clarified. The genes encoding some FAPs show regulation during flagellar assembly [13, 32, 77] and disassembly [44]. Twenty-one flagellar genes, also present in the *Chlamydomonas* proteome reference map [13], have been shown to directly regulate assembly and disassembly [44]. The expression profile of FAP12, for example, is similar to that of known flagellar structural components. Alternatively, genes encoding the less well characterized FAP277 and FAP280 exhibited unique regulation profiles, which are not characteristic of known flagellar structural genes. It has been argued [44] that microarray technology has the ability to predict genes involved in regulatory networks on the basis of similar expression profiles [111]. This is another example of post-genomics contribution to improve the understanding on what could explain, then, how the product of the FAP12 gene may, therefore, serve a structural role, whereas the products of FAP277 and -280 may play regulatory roles, perhaps in regulating flagellar length. FAP133 has been suggested to be a component of the IFT system [112] based on two previous evidences: i) it is encoded in *Chlamydomonas* by a single gene (upregulated upon deflagellation) [32]; and ii) it is readily extracted from the flagella when the membrane is disrupted. Such hypothesis was further supported by the examination that FAP133 is specifically depleted, together with other IFT components, from mutant flagella [112].

Recently, *C. reinhardtii* FAP221 was found to be homologous to the mammalian protein Pcdp1, a member of a protein complex that interacts with Ca2+-CaM and localizes to the C1d projection of the central apparatus [113]. Such results provided the first assignment of polypeptides to the C1d central projection, and have, thus, established a definitive and essential role for FAP221 in regulating motility.

Studies like these [32, 44, 112, 113] add information to start defining the interrelationship between the cellular and molecular networks regulating flagellar changes. We believe that only through global, parallel expression and high-throughput analyses of the genes and proteins associated with flagellar assembly and disassembly (such as provided by microarray analyses or RNA and protein expression profiles, e.g.,) it will be possible to dissect the intricacies of this complex organelle and to uncover fundamental regulatory mechanisms that are part of a whole-cell response to flagellar stimulation.

### 2.3 Flagellar Chaperones

Heat-shock proteins (HSPs) are molecular chaperones known to localize within cilia and flagella and also to be highly induced during flagellar regeneration (HSP70A and HSP90A), playing important roles in flagellar and ciliary assembly [10, 114]. The high complexity and the highly specialized, continuous turnover of flagella can be illustrated by the fact that genes encoding flagellar proteins typically are transcriptionally upregulated during organellar assembly [34, 78, 115]. Therefore, it is quite obvious to realize why chaperones are so widely distributed ciliary and flagellar component [32], potentially related to overall axonemal protein dynamics [114]. Note that HSP70A is an abundant cytoplasmic protein also present in flagella [114] and just one of seven *Chlamydomonas* Hsp70 family members [116]. Within flagella, wild-type HSP70A is distributed in a discontinuous, punctate fashion and concentrates in flagellar tips [114], very similar to that of the components of the IFT system [12]. 

RNA expression data can provide information about potential cilia/flagella-related genes that is complementary to direct proteomic approaches [32], which can reveal only intrinsic components of the flagellum. The so-called RNA transcriptional profiling approach is also complementary to comparative genomics approaches because it can reveal genes (e.g., tubulin) that are found in organisms lacking flagella but that nevertheless play important roles in flagellar assembly [32, 71, 107], Gene function discovery by RNA transcriptional profiling tends to be most effective at identifying genes responsible for the development of new structures, such as in development [117], rather than identifying catalytic functions such as enzyme activities, which are typically not modulated in abundance by varying RNA transcription [32, 118]. In the case of flagella, even though turnover entails continuous assembly at the tip, the steady-state turnover is sufficiently smaller than the initial assembly rate. A strategy of identifying flagellar genes was validated with basis on induction with results addressing RNA transcription levels of 61 known flagellar components (33 found to be strongly induced during regeneration) [32]. These included genes encoding IDAs and ODAs, RSPs, IFT components, regulatory proteins, cofactors of tubulin folding, such as CPN2 and eight subunits of the T-complex protein 1 tubulin-folding factor (also known as CCT and to be involved with ciliary assembly [119]. The elevated requirement for tubulin-folding chaperones in assembling a microtubule-based structure such as the flagellum likely explains the induction of these genes, supporting the idea that analysis of RNA transcription induction during flagellar assembly can identify genes involved in assembly that are not themselves flagellar components [32].

### 2.4 Novel Roles for Unexpected Flagellar Proteins

Members of the ARF (ADP-ribosylation factor) family in membrane trafficking have known homologs linked to human ciliary diseases [120]. In *C. reinhardtii*, ARFA1a mRNA abundance decreases slightly during flagellar disassembly, but clearly increases during assembly [44]. Scorpion, a zebrafish cystic kidney gene, is a small GTPase in the ARF family [13] necessary for ciliary assembly [121]. In addition, *C. elegans* ARL6, a member of the ARF-like (ARL) family of GTPases, is linked directly to BBS [122] ARL6 is specifically expressed in ciliated cells and undergoes bidirectional IFT [122]. On the basis of the link between ARL6 and IFT, a role was proposed in trafficking [122] not only in the cytosol, but also in the axoneme. The regulation of ARF expression [44] supports the possibility that ARF plays a similar role in *C. reinhardtii* IFT. In addition, it was also proposed that other conserved small GTPases, like ARL-13 and ARL-3, coordinate to regulate IFT and that perturbing this balance results in cilia deformation [123].

Two other proteins, a calcium-binding protein, calreticulin (CRT2) [124] and CALK, a *Chlamydomonas* aurora kinase [125], have also been linked to flagellar assembly and disassembly. A few works have demonstrated that the processes of flagellar excision, gene induction, and outgrowth are each independently regulated by calcium (Ca2+) [126]. CRT2 was shown to have decreased abundance of mRNA during flagellar disassembly, but increased CRT2 during assembly [44], whereas CALK has gained status as a crucial element in the cell's ability to regulate flagellar excision and disassembly by the demonstration [125] that it acts in an early step in both flagellar loss and disassembly and that its down-regulation correlates with down-regulation of its activity later in both assembly and disassembly. 

### 2.5 Posttranslational Modified Flagellar Proteins

Flagellar proteins, α- and β-tubulins, are known to undergo various posttranslational modifications, including phosphorylation, palmitoylation, tyrosination/detyrosination, Δ2 modification, acetylation, glutamylation, and glycylation [127]. Methylation of flagellar proteins, although a new observation with respect to flagellar dynamics [128], is not the only demonstration of posttranslational protein modification in flagella. For example, numerous phosphorylated proteins have been identified in *Chlamydomonas* flagella, including {alpha}-tubulin [127, 129], RSPs [130], ODA [131], and a number of membrane/matrix components [132]. Previous experiments have indicated that protein phosphorylation levels change with alterations in flagellar activity [133]; indeed, the flagellum was long known to contain >80 phosphoproteins [130]. Despite the importance of this posttranslational modification, the identity of many flagellar/ciliary phosphoproteins and the knowledge about their *in vivo* phosphorylation sites are still missing [18]. Boesger *et al.* (2009) [18] have used immobilized metal affinity chromatography (IMAC) to enrich phosphopeptides from purified flagella that were analyzed by mass spectrometry (MS) and they found 141 phosphorylated peptides that belong to 32 flagellar proteins. The authors present a flagellar phosphoproteome that includes different structural and motor proteins, kinases, proteins with protein interaction domains as well as many proteins whose functions are still unknown. Phosphoproteins can possess more than one phosphorylation site, and the phosphorylation status of these sites can fluctuate depending on the physiological conditions under which the cells are kept [134]. This leads to a great variety of phosphoproteins. In addition, the ratio of the phosphorylated to nonphosphorylated form of a protein can be very low. Although proteins can be identified down to the femtomole, and even attomole, level with modern MS, many phosphoproteins within a crude extract (especially those of cell signaling pathways) are not abundant enough to be unambiguously identified by MS. For this reason, enrichment of such proteins is often a prerequisite for efficient phosphoproteome analysis.

Phosphorylation has recently been shown to be important also in the control of flagellar length, as is IFT itself [107, 108]. Variations in flagellar length in *Chlamydomonas* have been correlated with the activity of a novel MAP kinase encoded by the LF4 gene [135], a NIMA-related kinase [136], and glycogen synthase kinase 3 [137], although the target proteins for these kinases have not yet been identified [126].

Axonemal tubulin undergoes several other modifications in addition to phosphorylation, including glycylation, acetylation, and polyglutamylation [127, 128]. Recent identification of tubulin-modifying enzymes, especially tubulin tyrosine ligase-like proteins, which perform tubulin glutamylation and glycylation, has demonstrated the importance of tubulin modifications for the assembly and functions of cilia and flagella [127].

Glycylation in ciliary axonemes [138], for example, is such an essential modification that in *Tetrahymena* it has been linked to a 9+0 and immobile axoneme [139]. Deacetylation and phosphorylation reactions are important in the disassembly of primary cilia, such as in the case of HDAC6, a tubulin deacetylase, that is activated by phosphorylation via CALK [125], what, in turn, promotes ciliary disassembly [140]. 

## 3 THE NANOSTRUCTURED FLAGELLUM MADE OF 650 PROTEINS

**Fig. (2):**
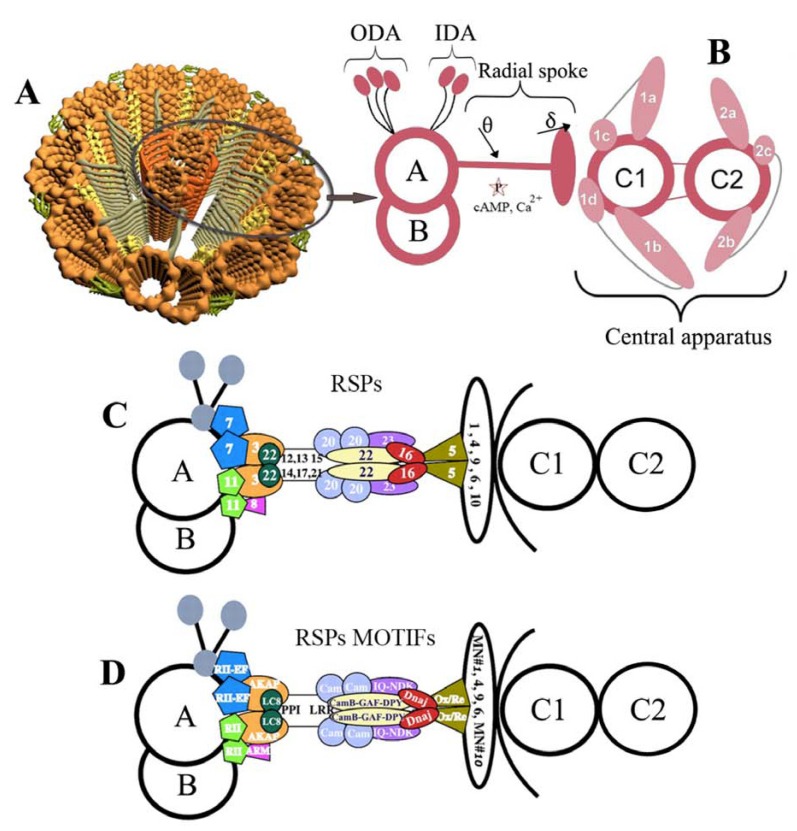
**Schematic illustration of axonemal sub-structures of the eukaryotic flagellum. Panel A,** an overview of its internal, axonemal architecture showing the 9+2 cannonical pattern of two inner, central pair (CP) microbules and nine outer microtubule doublets (in golden color), each named A-tubule and B-tubule. Note the presence of radial spokes (RS) and RS proteins (RSPs) as T-shaped structures (in gray) extending from the A-tubule of each doublet to the center of the axoneme (CP), as depicted in **Panel B,** illustrating the interconnected elements of RSPs, outer dynein arms (ODAs) in green and inner dynein arms (IDAs) in yellow, CP projections in orange. **Panel C** is the hypothetical “stalk” & “head” model of RSP as a mechanochemical transducer extending from the 9+2 and anchored near the base of the IDA, where the signal input includes calcium binding and/or mechanical strain induced by transient interaction of the spoke head with the central apparatus. Adapted from (and modified after) [113]. Since CaM anchored to the axoneme is a key calcium sensor, while central apparatus and RS are integral elements of calcium signaling pathway [218], four different CaM-interacting protein complexes have been localized: i) to the stalk, RSP2; ii) to the base of the spoke, FAP91; iii and iv) to CP projections, FAP101 and FAP221 [113], with three of these homologs being present in *Leishmania* genomes (CAC14327, CAB71185 and LmjF35.0290). Several models, including those proposed by [113], [148] and [218], are considered in **Panel D,** which stands for the probable locations of the RSPs, and in **Panel E,** which stands for their molecular modules relative to a CP microtubule (right) and an IDA on an outer doublet (left).

The eukaryotic flagellum is a biological nanomachine that is a self-contained mechanochemical oscillator and a force-producing organelle of motility [141] found in organisms as diverse as trypanosomes, green algae, and mammals. Although its 9+2 arrangement has been highly conserved through eukaryotic evolution, there are examples where this standard layout has been modified, including the "9+0" layout of primary cilia and the "9+9+2" of many insect sperm flagella [142]. In addition to this, flagella and cilia show a vast range of key substructures (elegantly visualized in sea urchin by Nicastro *et al.*, 2005 [143] and modeled in Fig. **2**), such as the inner (IDA) and outer dynein arms (ODA), and radial spokes [144-148]. Other discrete substructures are nexin links, bipartite bridges, beak-like projections, ponticuli, and other microtubule elaborations that are also essential for cilium/flagellum function. At the base of the eukaryotic flagellum lies a basal body (BB) or kinetosome, which is the microtubule-organizing center for flagellar microtubules. BBs are structurally identical to centrioles. Furthermore, the existence of extra-axonemal structures particular to groups of organisms, such as the paraflagellar rod (PFR) in trypanosomatids [60, 105] and the fibrous or rod-like structures in *Giardia lamblia* [149], contribute to an increase in the organelle complexity that never ceases to amaze us. 

### 3.1 Flagellar Dynamics of ATP and Energy Metabolism

As an engine of motility, and like other engines, the axoneme undergoes a cycle of linked events that harness the release of chemical energy to produce useful work. To understand the internal events in the beat cycle, it is essential that we understand the interaction of the forces from the primary dynein motor proteins with the structural components of the axoneme [141, 150]. We must recall that the demand from the dynein motors for ATP can be satisfied by the presence of discrete energy generating pathways organized specifically within the flagellar compartment. Importantly, the biochemical identity of such pathways reflects the environment in which the flagellum beats [reviewed by 37]. 

It has been shown that an ability to provide ATP along the length of the axoneme could be important for sperm motility [151], while now at least four (04) distinct mechanisms for flagellar energy-generating systems can be anticipated. 1) The phosphotransfer relay, in which ATP, generated through oxidative phosphorylation, is trafficked along the length of the axoneme by a creatine kinase-catalysed phosphocreatine shuttle. This mechanism is known to occur in sea urchin [151] and rooster sperms [152]. 2) A regular glycolytic pathway, in which mammalian sperms use glycolytic enzymes, such as hexokinase and glyceraldehyde-3-phosphate dehydrogenase, to swim within the microaerobic environment of the female reproductive tract. 3) A semi- or partial glycolytic pathway [153, 154], where *C. reinhardtii* flagella possess three enzymes of the lower half of the glycolytic pathway, which allow ATP production *in situ* from the glycolytic intermediate 3-phosphoglycerate. One of these enzymes, enolase, is linked to the axoneme through its association with the central pair protein CPC1, whereas the other two glycolytic enzymes, phosphoglycerate mutase and an unusual pyruvate kinase, are located in the membrane + matrix fraction. 4) A putative adenylate kinase-based flagellar energy-generating system [155, 156] in which most enzymes of the glycolytic pathway are compartmentalized within the peroxisomal matrix, giving rise to the classification of peroxisomes as glycosomes in trypanosomes [157]. 

### 3.2 The Axoneme

The canonical axoneme (9+2 arrangement) - the structure most widespread and almost certainly ancestral to all others [158, 159] is anchored at the proximal end by a basal body (BB) containing triplet microtubules in a 9+0 arrangement. Dynein arms are attached to the A-tubule of each doublet such that their motor head domains are in close proximity to the B-tubule of the neighboring doublet [4, 160]. Activation of the dynein motors causes a sliding force between adjacent doublets [161]. Because the microtubules are constrained at the BB and along their length, this force is translated into an axonemal bend, what has been called the fundamental force of axonemal motion [162]. 

The movements of flagella are driven by multiple species of dynein heavy chains (DHCs), which constitute IDAs and ODAs. In *Chlamydomonas*, 11 DHC proteins have been identified in the axoneme, but 14 genes encoding axonemal DHCs are present in the genome. Each previously unassigned DHC gene was assigned to a particular DHC protein and it was found that DHC3, DHC4 and DHC11 encode novel, relatively low abundance DHCs, being localized to the proximal region of the growing flagella [163].

#### A) The Central Pair Complex (CPC).

Numerous studies have indicated that the central apparatus (or the central pair of singlet microtubules with associated projections that is called the central pair complex, CPC) plays a significant role in regulating flagellar motility, yet little is known about how the central pair of microtubules or their associated projections assemble [164]. The presence of the CPC is a characteristic of motile flagella (albeit some types of motile cilia or flagella naturally devoid of a CPC have been reported), while several proteins specifically associated to CPC have been directly implicated in flagellum motility [165], such as the axonemal enolase, which is a subunit of the CPC1 protein, as mentioned before on the semi- or partial glycolytic pathway**.**

Earlier comparative genomics revealed *hydin*, the hydrocephalus inducing gene *hy3*, as highly conserved in flagellates, including *C. reinhardtii* and human ciliated cells. Mutations in *hydin* and in other genes encoding ciliary proteins are known to cause hydrocephalus in mice. Flagellar proteomes showed hydin in *C. reinhardtii* [13] and *T. brucei* [29]. Furthermore, hydin has recently been located directly to axonemal central apparatus, as a CPC protein [166]. Hydin homologs in several species of *Leishmania* share high similarity to *C.* *reinhardtii* and *Danio rerio* (zebrafish) hydin [167], as a predicted polypeptide of ~590 kD encoded by a single copy gene spanning ~16,686 bp, as opposed to ~540 kD and ~17,700 bp in *Chlamydomonas*. For instance, LmjF30.1820, annotated as a conserved hypothetical protein in *L. major* genome at GeneDB and an ortholog of Tb927.6.3150, has been proposed as a novel hydin by means of its conserved motifs, adenylate kinase and ASH domains, that are both present and believed to bind on two other *Chlamydomonas* CPC proteins, Cpc1 and Pf6 [167].

#### B) The Flagellar Tip Complex (FTP).

One of the striking observations along the axoneme is that IFT particles move from base to tip at a constant rate without pauses [128]. At the flagellar tip, IFT particles are remodeled [168, 169] and, then, begin transport back to the cell body. A biochemical screen, based on difference gel electrophoretic (DIGE) analysis of purified flagella, has identified proteins that localize to the tip of the flagellum [128]. This region (and the proteins comprising it) is now being called the flagellar tip complex (FTC) [128]. These authors have employed DIGE to compare the protein composition of full-length versus regenerating (i.e., short) flagella in an attempt to identify proteins whose abundance in flagella is uniformly increased during regeneration. Proteins in short flagella that increase in abundance relative to full-length flagella would be potential tip proteins. As a matter of fact, they have identified one protein, the cobalamin (vitamin B12) independent form of methionine synthase that catalyzes the conversion of homocysteine to methionine via transfer of a methyl group from 5-methyltetrahydrofolate (MetE; EC 2.1.1.14 [EC])[128]. MetE had been previously identified in *Chlamydomonas* as a protein whose gene transcription is upregulated in gametes [170], whereas it is also a member of the *Chlamydomonas* flagellar proteome [13]. MetE is not localized to the flagellar tip, but rather it is distributed along the length of the flagellum, whereas the amount of MetE is higher in regenerating flagella compared with control, full-length flagella [128]. Arguments on what could be the function of MetE in flagella are needed. It does catalyze the conversion of homocysteine to methionine, which is then converted to S-adenosyl methionine (SAM) by methionine adenosyltransferase (EC 2.5.1.6 [EC]), itself a member of the flagellar proteome, what could indicate a potential requirement for protein methylation during flagellar assembly or disassembly dynamics [128]. Protein methylation has long been recognized as an important nuclear event, as histone methylation plays a key role in chromatin structure and transcriptional control. Because cilia and flagella are resorbed before cell division [171, 172], the data reported by Schneider *et al.* (2008) [128] is the first one to link progression through the cell cycle to a requirement for protein methylation in the flagellum.

### 3.3 Extra-Axonemal Structures

Cilia and flagella can also exhibit various extra-axonemal elaborations, and although these are often restricted to specific lineages, there is evidence that some functions, such as metabolic specialization, provided by these diverse structures are conserved [155, 173]. Examples of such extra-axonemal elaborations include the fibrous or rod-like structures in the flagellum of the parasite *Giardia lamblia* [147], kinetoplastid protozoa [105, 174], and the fibrous sheath in mammalian sperm flagella [175, 176], along with extra sheaths of microtubules in insect sperm flagella [142]. 

#### A) The paraflagellar rod (PFR).

All kinetoplastids build a flagellum that contains an extra-axonemal structure termed the paraflagellar rod (PFR) [105, 162], which usually consists of a complex subdomain organization of proximal, intermediate, and distal domains as well as links to specific doublets of the axoneme and a structure known as the flagellum attachment zone (FAZ) by which the flagellum is attached to the cell body for much of its length [105, 177]. This large structure runs along the axoneme from its point of emergence from the flagellar pocket until its distal tip, and it is tightly linked to the axoneme via physical connections to microtubule doublets 4–7 [105]. The PFR is required for kinetoplastid cell motility [178, 179] and survival [29, 180-184], serving as a scaffold for metabolic and signaling enzymes [155, 185, 186].

Two major protein components of the PFR (PFR1 and PFR2) have been identified [187-191] along with several minor PFR protein components [155, 185, 186, 192, 193], as well as two PFR-specific adenylate kinases, designated ADKA and ADKB [155], which have an unusual N-terminal extension that is both necessary and sufficient to localize these proteins to the PFR [17]. 

There is evidence that calmodulin interacts directly with one of the major PFR components [186], whereas several PFR proteins recently described [17] do have PFAM motifs predicted as calmodulin- or calcium-binding domains, in accordance with a predicted role for this interaction. The presence of calmodulin and the calcium and calmodulin recognition domains in the PFR sub-proteome is indicative of a calcium-regulated system operating within the PFR [17].

Despite being described almost fifty years ago, PFR structure has remained enigmatic until a recent report [194] has shed light on a few features of PFR architecture. Recent findings in trypanosomes have demonstrated that individual structural elements of each PFR zone are interconnected to form a single superstructure, while in the intermediate zone, parallel wall-like laths run the length of the flagellum [194]. Therefore, PFR itself is comprised of overlapping laths organized into distinct zones that are connected through twisting elements at the zonal interfaces. The overall structure has an underlying 57nm repeating unit. Biomechanical properties inferred from PFR structure lead to a proposal that the PFR functions as a biomechanical spring that may store and transmit energy derived from axonemal beating [194].

#### B) The Flagellar Pocket.

 New evidences agitate the region that comprises a point after the flagellum exits in *T. brucei*, the flagellar pocket [195, 196]. The pocket is an asymmetric membranous ‘balloon' with two boundary structures. One of these - the collar - defines the flagellum exit point. The other defines the entry point of the flagellum into the pocket and consists of both an internal transitional fiber array and an external membrane collarette. A novel set of nine radial fibers has been recently described in the basal body (BB) proximal zone [197]. In addition to axoneme and PFR components, a significant amount of membrane is required to construct a flagellum [40], while vesicles are targeted to the base of the flagellar compartment to deliver both membranes and membrane proteins [78]. In trypanosomes, it is known that all trafficking takes place in the flagellar pocket, the only site for endocytosis and exocytosis [for a review see 198]. Results show that in the absence of a new flagellum, a flagellar pocket structure remains associated to the bald BB [99, 100]. A flagellar sleeve seems to extend from the BB region of mutant trypanosomes (IFT80RNAi-induced cells), passing through the neck of the pocket [99]. This tip would be maintained on the existing flagellum by the flagellar connector, a structure that holds the distal end of the new flagellum to the side of the old flagellum [99, 199].

#### C) Flagellar Membrane.

A conserved membrane protein of kinetoplastids, KMP-11, which has been localized to the flagellum and flagellar pocket [200, 201], is currently an exciting concern because of its immunological properties recently uncovered [202]. Another examination of the KMP-11 RNAi phenotype in *T. brucei* has suggested a role for this protein in regulating BB segregation with additional consequences for nuclear and cell division [203].

## 4 GETTING TO THE ROOT OF TRYPANOSOMATID FLAGELLUM BY MEANS OF ACTIN-INTERACTING PROTEINS (AIPS)

The flagellum plays a key role in motility and sensory reception in some eukaryotic pathogens, being essential for parasite migration, invasion and persistence on host tissues [204]. The contribution of locomotion/movement to virulence is well documented for bacterial and viral pathogens. In the case of trypanosomatid protozoan pathogens, e.g., *Trypanosoma spp*. and *Leishmania spp*., which mediate their motility through flagellum, the contribution of cell motility to host-pathogen interactions had been largely unexplored until the early 2000’s [98]. There were significant evidences of roles for the flagellum in the control of cell size, shape, polarity and division (cytokinesis) in several organisms, including trypanosomatids [11], but not as a direct element in pathogenesis. More recently, some reports have distinguished putative *Leishmania *flagellar virulence factors and their organization in gene families [101], as well as components of the IFT complex [102] and key flagellar actin-interacting proteins [103, 204, 205]. To survey genes and proteins that can be assigned to a flagellar role in trypanosomatid pathogenesis, a few research groups have applied computational biology and post-genomic tools in order to improve/refine the identification of flagellar elements in genomes, transcriptomes and proteomes [17, 29, 40, 79, 100, 101, 185, 196, 206-211].

With the advances of genome related research and the computational biology advent, post-genomics and bioinformatics analyses have fundamentally changed the nature of research strategies; there has been an explosion of new information on all types of proteins, including the actin-associated proteins, their regulation, their roles in signaling and also in flagellar assembly and disassembly. Some of these proteins have close homologs in both prokaryotic and eukaryotic systems, becoming clear that the mechanisms behind their functional roles might be essentially similar across divergent species. Bioinformatics analysis also intends to provide initial elements for guiding future *in vitro *studies, while these recent data provide a more detailed annotation of gene products. This is another point that will help to improve the current knowledge about flagellate organisms and their proteins of interest, such as flagellar actin and actin-related or –interacting proteins (ARPs or AIPs). We must recall that the driving force underlying internalization into the host cell is thought to involve both polymerization of parasite actin and actin motor-associated proteins. Investigations have undertaken comparative genomics and post-genomics in flagellate organisms to address their flagellar dynamics [79, 211, 212]. Since AIPs are actively involved in remodeling of the actin cytoskeleton (and the respective signaling mechanisms) via activation of other AIPs and microtubule-related activities, both processes directly involve cell motility and the eukaryotic flagellum (and a network of associated proteins). Therefore, clarifying the elements that play a role in such flagellar remodeling network might improve our understanding of how trypanosomatids establish a successful infection. 

Our own recent post-genomics work [101-103, 205, 206, 209] has focused upon flagellar metabolism in the pathogenic protozoan *Leishmania*. Results concerning these detailed sequence and structural analyses, performed on different data, turned out to unveil genes (and gene products) such as profilin, formin, katanin, coronin, cofilin, twinfilin, among others. These proteins have a common feature of actin-binding/interacting activity and might be involved in *Leishmania *intraflagellar pathways. *In vitro* and *in silico *examinations have helped in the secondary annotation of pathogen genomes, such as *Leishmania spp.*, an indirect contribution to a better understanding of the diseases they cause.

Proteins such as profilins are thought to regulate actin polymerization in response to extracellular signals, acting at a critical control point in signalling pathways initiated by events at the plasma membrane, and playing a crucial role in regulating the activity in the microfilament system and intracellular calcium levels [reviewed in 213]. The importance of profilins for normal cell proliferation and differentiation has been documented in genetic studies, showing that profilin gene disruption leads to grossly impaired growth, motility, and cytokinesis in single cells and embryonic lethality in multicellular organisms such as insects and mice [213]. Studies on profilins (regulators of cytoplasmic actin dynamics, binding to several nuclear proteins) have been performed on the sense that, although not yet experimentally characterized in flagellated protozoa, profilins might have a distinctive role on parasite flagellar dynamics and remodeling since they are also actually part of the flagellar proteome map reference. Their importance in the trypanosomatid IFT process can be greater than hinted at first; markedly if, in a near future, experimental *in vitro *work with profilins succeeds to prove that they actually function as hubs of a complex network of molecular interactions in the flagellum. Moreover, the subcellular localization of functional profilins [214] and their constant presence on flagellar proteomes [13, 30] provide additional evidences for specific roles in flagellar activities.

ADF/cofilins are ubiquitous actin dynamics-regulating proteins that have been mainly implicated in actin-based cell motility [215]. They are formed by a single folded domain, the ADF homology (ADF-H) domain, which is also found in other AIP families, including Abp1p, drebrins (a single ADF-H domain linked to another motif), twinfilin (a duplication of this domain is the reason for the name) and coactosin. The ADF/cofilins themselves vary in size from 113 to 168 amino acids, while the main actin-binding structure of the ADF/cofilins is the long alpha-helix starting, for example in human destrin, at Leu111 and terminating at Phe128. Most ADF/cofilins contain at least one nuclear-localization signal (NLS) close to the amino terminus. Trypanosomatids also contain a putative ADF/cofilin homologue [211, 215], as there are three sequences on *Leishmania* genomes that correspond to a cofilin-like gene in each species, *L. major*, *L. infantum* and *L. braziliensis* (LmjF29.0510, LinJ29_V3.0520 and LbrM29_V2.0450), one of them modeled to a 3D structure as can be seen on Fig. (**4**). An interesting and instructive sequence (and, by inference, structural) variance among compared cofilin sequences is revealed by few details in multiple alignments shown by Pacheco *et al.* (2009) [205] and also represented here in the Fig. (**4**). Nevertheless, the ADF/cofilin role in trypanosomatid flagellar motility remained largely unexplored until the ADF/cofilin gene was knocked out in *Leishmania* by targeted gene replacement and resultant mutants were completely immotile, short and stumpy, with reduced flagellar length and severely impaired beat [211]. In addition, the assembly of the paraflagellar rod was lost, vesicle-like structures were seen throughout the length of the flagellum and the state and distribution of actin were altered. The authors observed that episomal complementation of the gene restored normal morphology and flagellar function, what helped them to conclude that the actin dynamics-regulating protein ADF/cofilin plays a critical role in assembly and motility of the *Leishmania* flagellum [211, 215]. 

## 5 CURRENT PERSPECTIVES FOR NOVEL FLAGELLAR ROLES

Several recent studies have set out to determine the protein composition of the flagellum and demonstrated the existence of both an evolutionarily conserved core of flagellum proteins and a large number of lineage-restricted components [10, 13, 17, 26, 29, 210, 216]. Although these approaches provide an invaluable catalogue of the protein components of the flagellum (Table **1**), it has been argued that they provide only limited information on the substructural localization of proteins and do not address either the likely protein-protein interactions or the function of these proteins within the flagellum [17]. 

### 5.1 Recent Proteomic Complimentary Techniques

In this regard, the protein composition of some axonemal substructures, such as RSP complexes [148, 217], has been determined by direct isolation of these structures, and a number of complexes have been resolved by the use of co-immunoprecipitation of indicator proteins [218]. In addition, the localization and function of a number of flagellar proteins have been investigated by detailed analysis of mutant cell lines of *C. reinhardtii* exhibiting defined structural defects within the assembled axoneme. The early studies of Luck and Piperno employed 2D PAGE to compare protein profiles of purified flagella derived from *C. reinhardtii* mutants and wild type cells [129, 130, 219, 220], but these elegant works did not allow identification of the individual proteins within the profiles [17]. 

On the other hand, recent proteomic advances offer the opportunity to improve this identification, good examples being the comparative proteomic technique isotope coded affinity tagging [221], which has been used to identify components of the ODA [222], and the immobilized metal-ion affinity chromatography (IMAC), which is based on the presence of negatively charged phosphate groups and enriches for phosphorylated Ser, Thr, and Tyr [31, 134]. The first technique utilizes stable isotope tagging to quantify the relative concentration of proteins between two samples [17]. Additional comparative approaches include the utilization of 2D difference gel electrophoresis (DIGE, [223]) and isobaric tags for relative and absolute quantitation (iTRAQ; Applied Biosystems) to reveal protein components of flagellar structures via ablation by inducible RNA interference mutation [17]. These two complementary proteomic approaches, DIGE and iTRAQ, were used together with RNAi, establishing a mutant/proteomic combination as a powerful enabling approach for revealing dependences within subcohorts of the flagellar proteome, with 20 novel proteins identified as components of the PFR [17]. The authors have argued that the detected dependences might be due to interactions in the final PFR structure or a result of the process of transporting proteins to the flagellum.

Other resources such as quantitative structure–activity relationship (QSAR) methods, which are very useful in bioorganic and medicinal chemistry to discover small-sized drugs, may help to identify new targets, if applied to flagellar proteins, as recently studied with *Leishmania* dyneins [210]. Another current approach is to apply proteomics to the investigation of posttranslational modifications as phosphorylation, one of the key modifications of proteins, which is crucial in the control of many regulatory pathways, affecting protein function, activity, stability, localization, and interactions [134]. Therefore, information about the phosphoproteome (the proteome analysis of phosphoproteins) is extremely useful for understanding a variety of cellular processes, with several previously identified flagellar phosphoproteins of *C. reinhardtii*, such as the {alpha} heavy chain of ODA, RSPs [131, 134] and IC138, a WD repeat dynein intermediate chain [224], being validated through the latest flagellar phosphoproteome reported by [18].

### 5.2 Post-Genomic Complimentary Techniques

The maskless photolithographic DNA synthesis technology [32, 117, 225] is a means to construct high-density DNA oligonucleotide microarrays to represent exons from each strand of a given genome, as it has been done for the *Chlamydomonas* genome [32]. These authors measured the transcriptional activity for all of the *Chlamydomonas *exons, while arrays were probed with fluorescence-labeled cDNA, reverse-transcribed from total RNA isolated from cells that were grown for 30, 45, and 120 minutes after deflagellation [65], in very elegant experiments [32]. 

Microarray and genomics and proteomics techniques, plus libraries of expressed sequence tags (ESTs), in combination with digital differential display tools and publicly available gene expression and genome databases, are being currently used to identify and characterize novel flagellar and flagella-related proteins [28, 203], as illustrated by the large number of recently characterized proteins. The ability of bioinformatics and these aforementioned techniques to identify cilia and flagella-related genes has been documented several times [10, 13, 14, 26, 28, 32]. The power of such proteome approaches lies in the identification of novel components (Tables **1** and **2**) and modifications that have not been discovered before [31]. 

## 6 FINAL REMARKS

In quoting Gibbons & Grimstone (1960) [3] inspiring words about the eukaryotic flagellum: *“one cannot fail to be impressed by its extraordinary complexity…and…relative simplicity*”, we could not pick a better statement to close this review. Our own impression on this intriguing and never-ending surprising organelle is that it is on the beginning of its emergence to large audiences. Investigations on the functions of flagella-specific proteins (Tables **1** and **2**) will continue to enlighten the unique biological activities of the flagellum and future endeavors should further refine our knowledge of flagella and cilia at its designated cellular address. One of the most relevant discoveries in flagellum research, the IFT [82], has set the cornerstone for a new appreciation of cilia as antennae that sense fluid flow, fluid pressure, or ligands that facilitate intercellular signaling and can link specific molecular defects in this organelle to a host of human ciliopathies [226]. These ciliopathies are marked by an amazing diversity of clinical manifestations and an often complex genetic aetiology [227]. The green algae *Chlamydomonas* and its pair of flagella have taught us all a lot [82, 227]; and surely they will keep being essential for improving our comparative understanding of so many important events that comprise this unique organelle. Moreover, multicellular organisms such as mouse, zebrafish, *Xenopus*, *Caenorhabditis elegans* or *Drosophila*, and protists such as *Paramecium*, *Tetrahymena*, *Trypanosoma* and *Leishmania* each bring specific advantages to the study of flagellum/cilium biology [227]. For all that has already been discovered (and for all that yet remains to be clarified) about the eukaryotic flagellum, it is clear that it will make many more days as the current one giving name to this review.

## SUPPLEMENTARY MATERIALS

Supplementary material is available on the publishers web site along with the published article.

## Figures and Tables

**Fig. (1):**
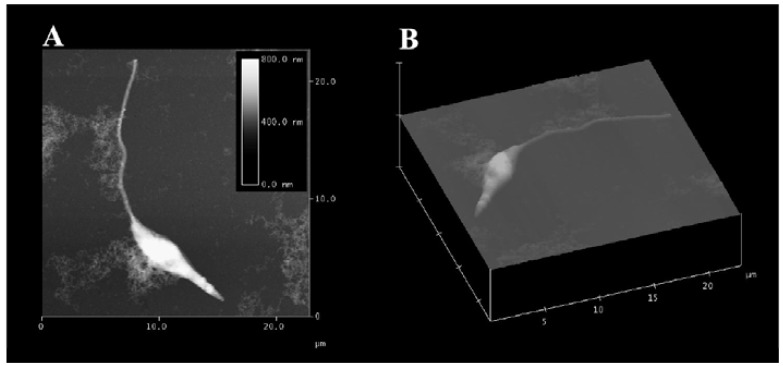
(**A**) Atomic force microscopy (AFM) contact mode image of an isolated promastigote form of *Leishmania chagasi* showing its elongated cell body with a single flagellum in surface topography. (**B**) Contact mode 3D view.

**Fig. (3):**
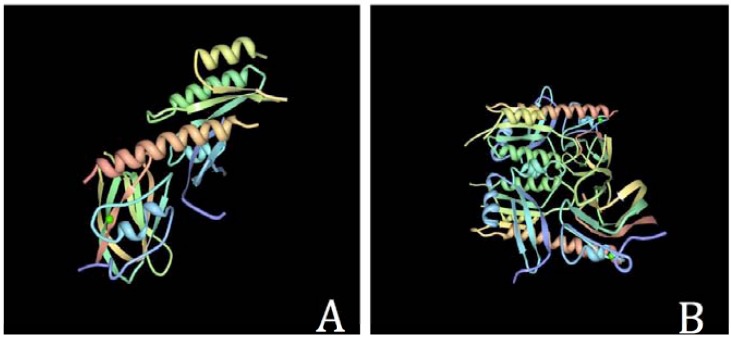
Three-dimensional structures of the first intraflagellar transport (IFT) proteins deposited at Protein Data Bank (PDB). The *Chlamydomonas reinhardtii* IFT complex 25/27 can be seen on panels A (PDB ID 2CY2) and B (2CY4) [110]. Images are viewed after PDB access modifications made in RCSB PDB Protein Worshop 3.9^®^.

**Fig. (4):**
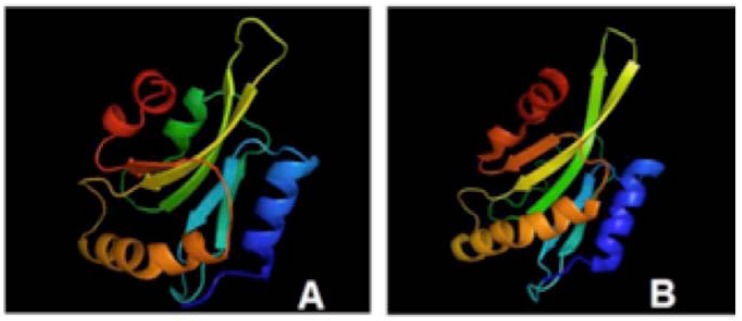
Three-dimensional structure of cofilin. **A**) A 3D model of *Leishmania infantum* cofilin after **B**) the PDB template 1QVP_A. A significantly well conserved display of secondary and tertiary structural features can be seen and easily correlated to the average 37% overall similarity between the two primary sequences. Both cofilins have a central mixed β-sheet, which is sandwiched between two pairs of α- helices. The highly conserved residues said to be important for protein stability and correct folding (Tyr64, Trp88, Pro90, and Tyr101, with the exception of Phe85) are present in all *Leishmania* cofilin sequences and shown in *L. infantum* modeled cofilin.

**Table 1.** List of Functional Categories of Eukaryotic Flagellar Proteins by Groups of Known Roles and Activities, With Number of Representatives. A Detailed View from the General Division in Six Major Groups (93 Signal Transduction Proteins; 87 Conserved Uncharacterized Proteins; 41 Motor Proteins; 39 Predicted Membrane Proteins; 09 Nucleotide Metabolism Proteins; 08 Glycolytic Enzymes + Malate Dehydrogenase) Made in Reference [13] was Modified Henceforth to Include Updated Information Obtained from References [10, 17, 18, 26, 28, 29, 30, 32, 38, 47, 73, 89, 90, 148]

Functional Category	Division		Numbers	Main Members
Motor Proteins (Sub-total 50)	Tubulins		05 (02 alpha and 03 beta tubulins)	C_30120; C_18960001 C_1320004; C_70002
	Kinesins		04 (02 FLA-Kinesin-II, 01 KLP - Kinesin-like protein and 01 KAP – Kinesin associated protein)	C_1880008, FLA10; C_160226, FLA8; C_50080,KLP1; C_620048, KAP
	Dyneins	-ODA	19	C_370072, ODA6;
		-IDAs	18	C_1310009, IDA5;
		-DHC/DRC	04	C_20225, DHC4;
Axonemal Proteins (Sub-total 17)	“General Axonemal”		10	C_60116; C_1020014 C_410060 HYD3,
	Central Pair Complex (CPC)		07	C_1580011, CPC1 C_60158; C_80166 C_40010
IFT Proteins (Sub-total 22)	IFT Complex A (07) IFT Complex B (15)		18th IFT particle, IFT25, whose complex with IFT27 (IFT25/IFT27) is the first crystal structure of an IFT member at PDB [110]	C_1510005 IFT20 C_640055 IFT140 C_170190 IFT172 C_410035 IFT72/74, C_320063, FAP232, IFT25, IFT22
Chaperones	Heat Shock (HSPs)		RSP stalk protein – member of HSP40 family	C_1340012; C_70195; C_730014; HSP40 C_110180, putative chaperonin
RSPs (Sub-total 26)	RSPs		26 (At least 05 spoke head and 16 spoke stalk proteins, besides others)	C_900027; C_83001; C_20323; C_490039 C_420011; C_60025 C_3230001
Predicted Membrane Proteins (Sub-total 39)	Flagellar Membrane		04	C_730054; C_73005; Flagella Membrane Glycoproteins FMG-1B and FMG-1A, C_1520018; C_40046
FAPs (Sub-total 271)	Several Predicted Roles		271 FAPs are a main source to be investigated, since they are likely to be important flagellar elements	C_240009, FAP158, C_1170006 FAP150, FAP221
Glycoproteins	Hydroxyproline-Rich Glycoproteins		06	C_200178; C_220001; C_3200003; C_200177; C_430092; FAP150
	Pherophorins		07	
Calcium-binding Proteins (CBP) (Sub-total 11)	Calcium-Transporting ATPases Calcium/Calmodulin-Dependent Protein Kinase Caltractin / Centrin		-	C_860007; C_370080; C_700061; C_800022 C_1500009 VFL2, Caltractin / Centrin, C_450071
Unnamed (Sub-total 30)	Coiled-coil Proteins (19); Leucine-Rich Repeat (04); EF-hand repeat (04); WD40 repeat (02); TPR-containing (01)		Flagellar Proteins with Key Motifs	-
Enzymes (Sub-total 27)	Dehydrogenases Isocitrate Lyase, Flavodoxins METE, MethionineSynthase		Glycolytic + Others, but not kinases	C_160138 Enolase C_280107;C_660012; C_1390004; C_2020016; C_1460023
Protein Kinases (Sub-total 20)	Pyruvate, Casein and Adenylate Kinases NIMA-related kinases		Fourteen proteins very similar to protein kinases	C_100034;C_1160048; C_60170; C_380089; C_70149; C_160119; C_2590004; C_450071
Others (Sub-total 108)	Ex: four proteins very similar to Pherophorins		Proteins Very Similar to Known Proteins	C_1550010; C_300008; C_10250; C_300007
Uncharacterized (Sub-total 78)	Conserved Uncharacterized		11	-
	Hypothetical Proteins		05 (Similarity with known motifs / domains)	-
	Simply Hypothetical Proteins		62	-

Proteins IDs are those used in the *Chlamydomonas* flagellar proteome [13] and they may be included in more than one category (e.g., a calcium-binding protein that is also an enzyme such as C_700061, Similar to Calcium Transporting ATPase). References used in the table: [10] Ostrowski *et al*., 2002; [13] Pazour *et al*., 2005; [17] Portman *et al*., 2009; [18] Boesger *et al*., 2009; [26] Smith *et al*., 2005; [28] McClintock *et al*., 2008; [29] Broadhead *et al*., 2006; [30] Jia *et al*., 2010; [32] Stolc *et al*., 2005; [38] Cao *et al*., 2006; [47] Li *et al*., 2004; [73] Liang *et al*., 2008; [80] Wang *et al*., 2006; [89] Follit *et al*., 2009; [90] Lechtreck *et al*., 2009; [136] Bradley *et al*., 2005; [148] Yang *et al*., 2006.

**Table 2.** List of Eukaryotic Flagellar/Ciliary Proteins Linked to Novel and/or Unanticipated Crucial Roles in Motility and Sensorial Functions/Dysfunctions. Each Protein is Displayed with its Given Name, Main Source Organism, Main Roles, Conserved Protein Motifs and Respective References

Protein Name	Local/Co-Expression/Organism	Function / Role	Motif / Pfam Domain	Ref.
**IFT25**	*Chlamydomonas* Component of the intraflagellar transport (IFT) complex B	Phosphoprotein that interacts with IFT27	-	Wang *et al.*, 2009
**IFT88**	Dermal papilla of developing hair follicles in human and murine	Signal reception of the sonic hedgehog (Shh) pathway/hair follicle cycling	TPR	Lehman *et al.*, 2009
**IFT172 Associated Protein (GASP-180)**	Component of IFT complex Cilia outgrowth in cells of the murine neuroepithelium	Required for proper function of the embryonic node, the early embryonic organizer and for formation of the head organizing center (the anterior mesendoderm, or AME) in mouse	WD40	Gorivodsky *et al.*, 2009
**RABL5 (small G protein; Rab-like 5)**	Traffic in sensory cilia of *Caenorhabditis elegans*; it co-localizes with IFT proteins at the basal body (BB) and in the flagellum matrix of *Trypanosoma brucei*	RABL5 participates in signalling processes but not in cilia construction	-	Adhiambo *et al.*, 2009
**RSP 44 (Meichroacidin)**	Human sperm	The 24th component of the radial spoke proteins (RSP) complex (now comprised of 26 proteins)	MORN	Shetty *et al.*, 2007
**Trypanosome Axonemal protein (TAX)-2**	*T. brucei* axoneme	Involved in processes linking the outer doublet microtubules and the central pair	DM10	Farr and Gull, 2009
**Myosin XXI**	Localized in the proximal region of *Leishmania* axoneme; partly associated with paraflagellar rod (PFR) proteins	Novel class of myosin, myosin XXI, in trypanosomatids, not associated with flagellar tubulins	-	Katta *et al.*, 2009
**Aurora kinase (AK)**	Localized to the paraflagellar dense rods of the anterior flagella in *Giardia lamblia* and pleiotropic localization	Reorganization and segregation of tubulin-containing structures in mitosis; First report of a signalling protein regulating cell division in *Giardia*	Serine/Threonine protein kinases, catalytic domain	David *et al.*, 2008
**Sporangin**	Co- localized to the flagella of the daughter cells of *Chlamydomonas *within the sporangial cell wall	First report of sporangin as a flagellar protein	Peptidase S8	Kubo *et al.*, 2009
**AP58 (ODA-binding protein)**	Tetratricopeptide repeat (TPR) motif-containing protein of sea urchin sperm axonemes	New member of ODA (outer dynein arm)-binding proteins	TPR	Ogawa K, Inaba, 2006
**Beta-Arrestins**	Human primary cilium / kinesin motor protein Kif3A; localized to the centrosome	Regulation of seven-transmembrane-spanning receptors (7TMRs); modulate the desensitization and trafficking of 7TMRs; development, cellular migration, and metastasis.	Arrestin_C	Kovacs *et al.*, 2008; Buchanan & DuBois, 2006
**THM1**	Mouse inner medullary collecting duct cells expressing an IFT88-enhanced yellow fluorescent protein fusion, which localizes to cilia.	Shh signal transduction TPR-containing Hedgehog Modulator-1 (THM1) protein	TPR	Tran *et al.*, 2008
**Spa17**	Sperm autoantigenic protein 17 is present in human and murine olfactory cilia; localized to sperm flagella and cilia of the respiratory tract and reproductive organs	Involvement in the binding of sperm to the zona pellucida of the oocyte and in additional cell-cell adhesion functions such as immune cell migration and metastasis	IQ calmodulin-binding motif	Lea *et al.*, 2004 McClintock *et al.*, 2008
**Annexin XX1 (annexin E1)**	Identical to alpha14-giardin; specifically localized to the flagella and to the median body of *Giardia lamblia* trophozoites	Adheres to microtubules of the flagella via self-assembly that may be regulated by Ser/Thr-phosphorylation.	ANX	Szkodowska *et al.*, 2002; Vahrmann *et al.*, 2009
*Giardia*** Axoneme Associated Protein (GASP-180)**	A new class of head-stalk proteins with a series of ankyrin repeats followed by a lengthy coiled-coil domain in *Giardia lamblia* flagella	A strong candidate to participate in control of flagellar activity	Ankyrin repeats and Coiled-coil domains	Elmendorf *et al.*, 2005
**Ftm (Rpgrip1l)**	Human and murine ciliary basal body and centrosome	A novel component for cilium-related Hh signalling / Sonic Hedgehog (Shh) signaling	Protein of unknown function (DUF3250) and a Myosin_tail_1	Delous *et al.*, 2007; Vierkotten *et al.*, 2007
**MAP kinases**	Control of flagellum length in the promastigote stage of *Leishmania*.	Putative Sensory role	S_TKc	Rotureau *et al.*, 2009
**(TPPP/p25)**	A brain-specific protein, expressed in human oligodendrocytes; glial and neuronal inclusions	TPPP orthologs are among the only 16 genes found in all ciliated organisms; Potential function as MAPs	-	Orosz and Ovádi, 2008
**TbLRTP**	Localizes to distal zones of BB; a trypanosomal protein with high similarity to a mammalian testis-specific protein of unknown function; BB duplication and flagellum biogenesis	Excess TbLRTP suppresses new flagellum assembly, while reduction of TbLRTP protein levels often results in the biogenesis of additional flagellar axonemes and intracellular PFR	Leucine-rich repeats and a Coiled-coil domain	Morgan *et al.*, 2005
**Hippi (HIP-1 Protein Interactor)**	Motile monocilia normally present at the surface of the embryonic node a dual role for Hippi in cilia assembly and Shh signaling during development/subtypes of malignant tumors	An adapter protein that mediates pro-apoptotic signaling from poly-glutamine-expanded huntingtin, an established cause of Huntington disease, to the extrinsic cell death pathway/interact with BLOC1S2 (protein is widely expressed in normal tissue as well as in malignant tumors)/ interaction with HIPPI and its pro-apoptotic activity, BLOC1S2 might play an important functional role in cancer and neurodegenerative diseases.	Coiled-coil	Houde *et al.*, 2006; Gdynia *et al.*, 2008
**IFT122/WDR10**	Primary cilia /Mouse (Mammals)	Coordinated movement of macromolecular cargo from the BB to the cilium tip and back/ Shh signaling/ ciliogenesis and Shh/Gli3 signaling	WD40	Cortellino *et al.*, 2009
**IFT20**	Cilium and centrosome/ anchored to the Golgi complex by the golgin protein GMAP210/Trip11/IFT particle is localized to the Golgi complex/mice/	Sorting proteins to the ciliary membrane	Coiled-coil protein	Follit *et al.*, 2008
**Kinesin-2 heterotrimeric complex**	Component of flagellar assembly (localized to both cytoplasmic and membrane-bound regions of axonemes)/ *Giardia intestinalis*	Anterograde movement of proteinaceous rafts along the outer doublet of axonemes in IFT	Cap-Gly domain	Hoeng *et al.*, 2008
**Elipsa protein (coiled-coil polypeptide)**	Ciliary component of IFT particles/ *Caenorhabditis elegans*	Ciliogenesis/ interacts genetically with Rabaptin5, a well-studied regulator of endocytosis, which in turn interacts with Rab8, a small GTPase, known to localize to cilia.	Microtubule-binding protein MIP-T3	Omori *et al.*, 2008
**NPHP1 and NPHP4 (nephrocystin-1 and nephrocystin-4)**	Basal bodies or ciliary transition zones (TZs)/ *Chlamydomonas reinhardtii*, *Caenorhabditis elegans*, and mammals	Unknown function in this location (BB or ciliary TZs) / globally regulate ciliary access of the IFT machinery, axonemal structural components, and signaling molecules,	Coiled-coil	Jauregui *et al.*, 2008
**FAP221/ Human Pcdp**	Cilia and flagella/*Chlamydomonas reinhardtii *Flagellar-Associated Protein (FAP) and Human Primary Ciliary Dyskinesia Protein 1 (Pcdp1)	FAP221 and mammalian Pcdp1 are central pair proteins that specifically bind CaM in high [Ca2+]/ essential for control of ciliary motility	CaM	DiPetrillo & Smith, 2010
**TbSAXO (MAP6-related protein – SAXO proteins)**	Cilia and flagella`s Microtubules/ *Trypanosoma brucei*	Axonemal protein that plays a role in flagellum motility (microtubule stabilizing protein); *T. brucei* STOP Axonemal protein) is the first MAP6-related protein identified in a protozoan.	Mn domain	Dacheux *et al.*, 2012
***Leishmania* ISPs (Inhibitors of Serine Peptidases)**	Localized along the flagellum/*Leishmania spp.; *ecotin-like natural peptide inhibitors of trypsin-family serine peptidases	Primary role in flagellar homeostasis, disruption of which affects differentiation and flagellar pocket dynamics.	ISP domain	Morrison *et al.*, 2012
**AKAP3 and CABYR ((calcium-binding tyrosine phosphorylation regulated)**	Human sperm proteins, Fibrous Sheath Protein 95 (FSP95), also known as AKAP3, and CABYR appear to associate in high molecular weight multi-protein complexes	Regulate the flagella through energy supply and movements	A Kinase Anchoring domain, AKAP	Naaby-Hansen, 2012
**Polo-like kinases (TbPlk)**	Localizes on BB, which nucleates the flagellum, and then successively localizes to a series of cytoskeletal structures and localizes to the flagellum attachment zone (FAZ)/*Trypanosoma brucei *and* Saccharomyces cerevisiae*	BB segregation and blocks the duplication of the regulators that position the flagellum (biogenesis)/ regulates only cytokinesis in *T. brucei*	S-TKc	Ikeda & Graffenried, 2012; Sun & Wang, 2012
**MNS1 (Meiosis-specific nuclear structural protein 1)**	Localizes to sperm flagella (is an integral component of flagella) in Mice/ also present in ciliary proteome of human bronchial epithelial cells	MNS1 is a novel and integral component of sperm flagella. Unknown function, but is essential for spermiogenesis, the assembly of sperm flagella, and motile ciliary functions	Coiled-coil	Ostrowski*et al.*, 2002; Zhou *et al.*, 2012
**GPCRs (G-protein coupled receptors)**	Cilia (ciliary assembly and disassembly)/*Chlamydomonas reinhardtii*	Ciliary formation, resorption, and length maintenance	-	Avasthi *et al.*, 2012
**TbMORN1, TbLRRP1, TbCentrin2, and TbCentrin4**	Localizes on trypanosome bilobe and basal bodies/*Trypanosome brucei* -	Composes the trypanosome bilobe (a cytoskeletal structure of unclear function)	Conserved C-terminal domain	Esson *et al.*, 2012
**HASPB (hydrophilic acylated surface protein B)**	Flagellum/*Leishmania*	Dual function protein that is shed by the infective metacyclic (retained internally once *Leishmania* are taken up by macrophages)	Eferin C-terminal domain-like	Maclean *et al.*, 2012
**Rab11-Rab8 cascade**	Cilia membrane assembly	Fundamental proteins required for the assembly of the microtubule-based backbone of cilia (ciliogenesis)	RAB	Qin, 2012
**TbVAP (*T. brucei* VAMP-associated protein)**	Associated with the flagellar pocket/*trypanosome brucei*	Maintenance of sub-populations of the endoplasmic reticulum associated with the FAZ and the flagellar pocket. VAMP-associated proteins (VAPs) are integral endoplasmatic reticulum (ER) membrane proteins (or vesicle-associated membrane proteins – VAMPs) whose mutation in humans has been linked to familial motor neuron disease.	MSP (Major sperm protein) and trans-membrane domain (TMD) domains;	Lacomble *et al.*, 2011
**MCA4 (Metacaspase4)**	Flagellar membrane via dual myristoylation-palmitoylation/ *T. brucei*	Multiple metacaspases in *T. brucei* form a membrane-associated proteolytic cascade to generate a pseudopeptidase virulence factor	CASs superfamily domain (a Caspase, interleukin-1 beta converting enzyme (ICE) homolog;	Proto *et al.*, 2011
**CRC70 (*Chlamydomonas* procentriole protein)**	Centriole and basal body protein / *Chlamydomonas reinhardtii*	Role in the accumulation of centriole proteins/ conserved protein family and functions as a scaffold for the assembly of the centriole precursor	Cep70	Shiratsuchi *et al.*, 2011
**H49/calpain (calpain-like cysteine peptidase family)**	Repetitive cytoskeletal protein, located along the flagellum attachment zone adjacent to the cell body/*Trypanosome brucei*	Structural role, attached to the flagellum by connecting the subpellicular microtubule array to it	H49	Galetovic *et al.*, 2011
**KH.C2.771 (Similar to the protofilament ribbon protein in axonemes)**	Sperm flagella - Axonemes/*Ciona intestinales*	Involved in Ca2+-dependent regulation of sperm motility	EF-hand domain	Lin *et al.*, 2011
**KH.L18.74 (EF-hand/ DM10 domain containing protein)**	Sperm flagella - Axonemes/*Ciona intestinales*	Involved in Ca2+-dependent regulation of sperm motility	EF-hand domain	Lin *et al.*, 2011
**KH.C7.450 (EF-hand/ DM10 domain containing protein)**	Sperm flagella - Axonemes/*Ciona intestinales*	Involved in Ca2+-dependent regulation of sperm motility	EF-hand domain	Lin *et al.*, 2011
**KH.C8.833 (EF-hand domain containing family member B)**	Sperm flagella - Axonemes/*Ciona intestinales*	Involved in Ca2+-dependent regulation of sperm motility	EF-hand domain	Lin *et al.*, 2011
**Tektin proteins (Tektin 1-5)**	Flagella / Rat spermatozoa	Involved in the stability and structural complexity of flagella	Coiled-coil domain	Takigushi *et al.*, 2011
**Qilin**	*Danio rerio*; cillium	Novel gene important in the pathogenesis of kidney cysts in zebrafish; qilin interacts with multiple intraflagellar transport (IFT) complex B genes; similar role as IFT complex B proteins in cilia assembly, maintenance and kidney development in zebrafish.	Coiled-coil and aspartic acid rich domains	Li and Sun, 2011
**α- and β-tubulins**	Cilia and flagella	Modifications for the assembly and functions of cilia and flagella	Tubulin Tubuli_C Coiled-coil	Konno *et al.*, 2012
**FAP54, FAP46, FAP74, FAP221/Pcdp1**	Associated with the central pair of microtubules/*Chlamydomonas*	Role in the control of flagellar motility	-	Brown *et al.*, 2012
**MARCH10a (membrane-associated RING-CH 10)**	Localized along the microtubules, as a microtubule-associated E3 ubiquitin ligase	Directly associated with microtubules/involved in spermiogenesis by regulating the formation and maintenance of the flagella in developing spermatids	-	Iyengar *et al.*, 2011
**ADF/cofilin**	Flagellum/*Leishmania*	Cell Motility	Cofilin_ADF	Kumar *et al.*, 2012
**Dynamin 3**	Associated with structures termed tubulobulbar complexes that internalize intact intercellular junctions/male Sprague–Dawley rats	Involved with tubulobulbar morphogenesis/function to stabilize the base of the tail or serve as a reservoir for use during or after fertilization (sperm cells).	DYNc PH GED	Vaid *et al.*, 2007
**FCaBP (flagellar calcium-binding protein)**	Targeted to the flagellar membrane/*Trypanosoma cruzi*	Regulates flagellar function and assembly	EF-hand domain	Wingard, *et al.*, 2008
**IFT 25/27 complex**	Cilium and flagellum/*Chlamydomonas reinhardtii*	Functions in motility, sensory reception and signalling	RAS Domain	Bhogaraju, *et al.*, 2011
**IFT70/ DYF-1 (IFT particle complex B)**	Flagellum within the IFT machinery /*Chlamydomonas reinhardtii*	Activator for an anterograde motor OSM-3 of IFT/function of IFT in building the flagellum	TPR	Fan, *et al.*, 2010
**CMUB116**	Sperm flagella of the ascidian/CMUB116 appears to be tightly associated with MORN40 and located at the stalk of radial spokes/*Ciona intestinalis*	Regulatory mechanism of sperm radial spokes in flagellar motility; the 26th component of RSP complex	Ubiquitin domain IQ motif	Satouh & Inaba, 2009

Proteins IDs are those used in the Chlamydomonas flagellar proteome [13] and they may be included in more than one category (e.g., a calcium-binding protein that is also an enzyme such as C_700061, Similar to Calcium Transporting ATPase). References used in the table are either listed in the manuscript or listed in the Supplementary Reference list provided.

## Footnotes

1 *Eukaryotic flagella are here discerned from their prokaryotic counterparts because they are completely different in structure and in evolutionary origin. The bacterial, archaeal, and eukaryotic flagella have in common the fact that they all project from the cell and wiggle to produce propulsion*.

## ABBREVIATIONS

PFR: = Paraflagellar Rod
IFT: = Intraflagelar Transport
FTC: = Flagellar Tip Complex
IDA: = Inner Dynein Arm
ODA: = Outer Dynein Arm
RSP: = Radial Spoke Protein
FAZ: = Flagellum Attachment Zone
CP: = Central Pair
CPC: = Central Pair Complex
BB: = Basal Body
ARP: = Actin-Related Protein
AIP: = Actin–Interacting Protein
DHCs: = Dynein Heavy Chains
HSPs: = Heat-Shock Proteins
MS: = Mass Spectrometry
MS/MS: = Tandem MS
DIGE: = Difference Gel Electrophoresis
iTRAQ: = Isobaric Tags for Relative and Absolute Quantitation
PIPES: = 1,4-piperazinediethanesulfonic Acid
MALDI: = Matrix-Assisted Laser Desorption Ionization
TOF: = Time-of-Flight
HPLC: = High Pressure Liquid Chromatography
PCR: = Polymerase Chain Reaction
qRT–PCR: = Quantitative Real-Time Polymerase Chain Reaction
RNAi: = RNA Interference
ESTs: = Expressed Sequence Tags
PI3K: = Phosphatidylinositol 3-Kinase
QSAR: = Quantitative Structure–Activity Relationship
CID: = Collision Induced Dissociation
PDK: = Polycystic Kidney Disease
BBS: = Bardet-Biedl Syndrome
SAM: = S-Adenosyl Methionine
MetE: = Cobalamin (Vitamin B12) Independent Form of Methionine Synthase
Shh: = Sonic Hedgehog (Shh) Signaling
LRR: = Leucine-Rich Repeat
ORF: = Open Reading Frame
CALK: = *Chlamydomonas* Aurora Kinase
ARF: = ADP-Ribosylation Factor
PDB: = Protein Data Bank
