# Supplementary material for: The Eukaryotic Flagellum Makes the Day: Novel and Unforeseen Roles Uncovered After Post-Genomics and Proteomics Data
Source: Curr Protein Pept Sci. 2012 Sep;13(6):524–46. doi: 10.2174/138920312803582951 (PMC3499766; doi:10.2174/138920312803582951)
Supplement: Supplementary file 1 — Supplementary material is available on the publishers web site along with the published article. [file CPPS-13-524_SD1.pdf]

SUPPLEMENTARY REFERENCES (THOSE APPEARING ONLY ON TABLES AND FIGURES, BUT NOT ON THE TEXT)

[SR1] Avasthi, P.; Marley, A.; Lin, H.; Gregori-Puigjane, E.; Shoichet, B.K.; von Zastrow, M.; Marshall, W.F. A Chemical Screen Identifies Class A G-Protein Coupled Receptors As Regulators of Cilia. *ACS Chem. Biol*., **2012**, Mar 8. [Epub ahead of print]

[SR2] Bhogaraju, S.; Taschner, M.; Morawetz, M.; Basquin, C.; Lorentzen, E. Crystal structure of the intraflagellar transport complex 25/27. Embo. J., **2011**, *10,* 1907–1918.

[SR3] Brown, J.M.; Dipetrillo, C.G.; Smith, E.F.; Witman, G.B. A FAP46 mutant provides new insights into the function and assembly of the C1d complex of the ciliary central apparatus. *J. Cell. Sci*., **2012**, May 8. [Epub ahead of print]

[SR4] Buchanan, F.G.; DuBois, R.N. Emerging roles of β-arrestins. *Cell Cycle*, **2006**, *5*, 2060-2063.

[SR5] Cortellino, S.; Wang, C.; Wang, B.; Bassi, M.R.; Caretti, E.; Champeval, D.; Calmont, A.; Jarnik, M.; Burch, J.; Zaret, K.S.; Larue, L.; Bellacosa, A. Defective ciliogenesis, embryonic lethality and severe impairment of the Sonic Hedgehog pathway caused by inactivation of the mouse complex A intraflagellar transport gene Ift122/Wdr10, partially overlapping with the DNA repair gene Med1/Mbd4. *Dev. Biol.,* **2009**, *325*, 225–237.

[SR6] Dacheux, D.; Landrein, N.; Thonnus, M.; Gilbert, G.; Sahin, A.; et al. A MAP6-Related Protein Is Present in Protozoa and Is Involved in Flagellum Motility. *PLoS One*, **2012**, *2*, e31344. doi:10.1371/journal.pone.0031344.

[SR7] Deligianni, E.; Morgan, R.N.; Bertuccini, L.; Kooij, T.W.; Laforge, A.; Nahar, C.; Poulakakis, N.; Schüler, H.; Louis, C.; Matuschewski, K.; Siden-Kiamos, I. Critical role for a stage-specific actin in male exflagellation of the malaria parasite. *Cell Microbiol*., **2011**, *11*, 1714-30.

[SR8] Esson, H.J.; Morriswood, B.; Yavuz, S.; Vidilaseris, K.; Dong, G.; Warren, G. Morphology of the trypanosome bilobe, a novel cytoskeletal structure. *Eukaryot. Cell*, **2012**, Feb 10. [Epub ahead of print]

[SR9] Farr, H.; Gull, K. Functional studies of an evolutionarily conserved, cytochrome b5 domain protein reveal a specific role in axonemal organisation and the general phenomenon of post-division axonemal growth in trypanosomes Cell Motil. *Cytoskeleton*, **2009**, *66*, 24–35.

[SR10] Follit, J.A.; SanAgustin, J.T.; Xu, F.; Jonassen, J.; Pazour, G.J. The golgin GMAP210/TRIP11 anchors IFT20 to the Golgi complex. *PLoS Genetics*, **2008***, 4,* e1000315*,* Epub 2008.

[SR11] Galetović, A.; Souza, R.T.; Santos, M.R.; Cordero, E.M.; Bastos, I.M.; Santana, J.M.; Ruiz, J.C.; Lima, F.M.; Marini, M.M.; Mortara, R.A.; da Silveira, J.F. The repetitive cytoskeletal protein H49 of Trypanosoma cruzi is a calpain-like protein located at the flagellum attachment zone. *PLoS One*, **2011**, *1*, e27634. Epub 2011 Nov 11.

[SR12] Gdynia, G.; Lehmann-Koch, J.; Sieber, S.; Tagscherer, K.E.; Fassl, A.; Zentgraf, H.; Matsuzawa, S.; Reed, J.C.; Roth, W. BLOC1S2 interacts with the HIPPI protein and sensitizes NCH89 glioblastoma cells to apoptosis. *Apoptosis*, **2008**, *13*, 437–447.

[SR13] Hoeng, J.C.; Dawson, S.C.; House, S.A.; Sagolla, M.S.; Pham, J.K.; Mancuso, J.J.; Lowe, J.; Cande, W. Z. High-resolution crystal structure and in vivo function of a kinesin-2 homologue in *Giardia intestinalis*. *Mol. Biol. Cell*, **2008**, *19*, 3124-3137.

[SR14] Ikeda, K.N.; de Graffenried, C.L. Polo-like kinase is necessary for flagellum inheritance in *Trypanosoma brucei*. *J. Cell Sci*., **2012**, Mar 16. [Epub ahead of print]

[SR15] Iyengar, P.V.; Hirota, T.; Hirose, S.; Nakamura, N. Membrane-associated RING-CH 10 (MARCH10 protein) is a microtubule-associated E3 ubiquitin ligase of the spermatid flagella. *J. Biol. Chem*., **2011**, *45*, 39082-90.

[SR16] Jauregui, A.R.; Nguyen, K.C.; Hall, D.H.; Barr, M.M. The *Caenorhabditis elegans* nephrocystins act as global modifiers of cilium structure. *J. Cell Biol.*, **2008**, *180*, 973–988.

[SR17] Konno, A.; Setou, M.; Ikegami, K.; Ciliary and flagellar structure and function-their regulations by posttranslational modifications of axonemal tubulin. *Int. Rev. Cell Mol. Biol*., **2012**, *294*, 133-70.

[SR18] Kumar, G.; Srivastava, R.; Mitra, K.; Sahasrabuddhe, A.A.; Gupta, C.M. Over-expression of S4D mutant of Leishmania ADF/cofilin impairs flagellum assembly by affecting actin dynamics. *Eukaryot. Cell*, **2012**, Apr 6. [Epub ahead of print]

[SR19] Lacomble, S.; Vaughan, S.; Deghelt, M.; Moreira-Leite, F.F.; Gull, K. A *Trypanosoma brucei* Protein Required for Maintenance of the Flagellum Attachment Zone and Flagellar Pocket ER Domains. *Protist*, **2011**, Dec 18. [Epub ahead of print].

[SR20] Li, D.; Yu, W.; Liu, M. Regulation of KiSS1 gene expression. *Peptides*, **2009**, 30, 130–8.

[SR21] Lin, J.; Tritschler, D.; Song, K.; Barber, C.F.; Cobb, J.S.; Porter, M.E.; Nicastro, D. Building blocks of the nexin-dynein regulatory complex in *Chlamydomonas* flagella. *J. Biol. Chem*., **2011**, *33*, 29175-91.

[SR22] Maclean, L.M.; O'Toole, P.J.; Stark, M.; Marrison, J.; Seelenmeyer, C.; Nickel, W.; Smith, D.F. Trafficking and release of Leishmania metacyclic HASPB on macrophage invasion. *Cell Microbiol*., **2012**, *5*, 740-61.

[SR23] Morrison, L.S.; Goundry, A.; Faria, M.S.; Tetley, L.; Eschenlauer, S.C.; Westrop, G.D.; Dostalova, A.; Volf, P.; Coombs, G.H.; Lima, A.P.C.A.; Mottram, J.C. Ecotin-like serine peptidase inhibitor ISP1 of *Leishmania major* plays a role in flagellar pocket dynamics and promastigote differentiation. *Cellular Microbiology*, **2012**, doi: 10.1111/j.1462-5822.2012.01798.x.

[SR24] Naaby-Hansen S. Functional and immunological analysis of the human sperm proteome. *Dan. Med. J.*, **2012**, *4*, B4414.

[SR25] Omori, Y.; Zhao, C.; Saras, A.; Mukhopadhyay, S.; Kim, W.; Furukawa, T.; Sengupta, P.; Veraksa, A.; Malicki, J. Elipsa is an early determinant of ciliogenesis that links the IFT particle to membrane-associated small GTPase Rab8. *Nat. Cell Biol*., **2008**, *4*, 437-44.

[SR26] Proto, W.R.; Castanys-Munoz, E.; Black, A.; Tetley, L.; Moss, C.X.; Juliano, L.; Coombs, G.H.; Mottram, J.C. *Trypanosoma brucei* metacaspase 4 is a pseudopeptidase and a virulence factor. *J. Biol. Chem*., **2011**, *46*, 39914-25.

[SR27] Qin, H. Regulation of intraflagellar transport and ciliogenesis by small G proteins. *Int. Rev. Cell Mol. Biol*., **2012**, *293*, 149-68.

[SR28] Reynolds, R.M.; Logie, J.J.; Roseweir, A.K.; McKnight, A.J.; Millar, R.P. A role for kisspeptins in pregnancy: facts and speculations. *Reproduction*, **2009**, *138*, 1–7.

[SR29] Satouh, Y.; Inaba, K. Proteomic characterization of sperm radial spokes identifies a novel spoke protein with an ubiquitin domain. *FEBS Lett*, **2009**, *583*, 2201-2207.

[SR30] Shetty, J.; Klotz, K.L.; Wolkowicz, M.J.; Flickinger, C.J.; Herr, J.C. Radial spoke protein 44 (human meichroacidin) is an axonemal alloantigen of sperm and cilia. *Gene*, **2007**, *396*, 93-107.

[SR31] Shiratsuchi, G.; Kamiya, R.; Hirono, M. Scaffolding function of the Chlamydomonas procentriole protein CRC70, a member of the conserved Cep70 family. *J. Cell Sci*., **2011**, *17*, 2964-75.

[SR32] Sun, L.; Wang, C.C. The Structural Basis of Localizing Polo-Like Kinase to the Flagellum Attachment Zone in *Trypanosoma brucei*. *PLoS One*, **2011**, *11*, e27303.

[SR33] Takiguchi, H.; Murayama, E.; Kaneko, T.; Kurio, H.; Toshimori, K.; Iida, H. Characterization and subcellular localization of Tektin 3 in rat spermatozoa. *Mol. Reprod. Dev*., **2011**, *8*, 611-20.

[SR34] Vaid, K.S.; Guttman, J.A.; Babyak, N.; Deng, W.; McNiven, M.A.; Mochizuki, N.; Finlay, B.B.; Vogl, A.W. The role of dynamin 3 in the testis. *J. Cell. Physiol*., **2007**, *3*, 644-54.

[SR35] Wang, M.; Gheiratmand, L.; He, C.Y. An interplay between Centrin2 and Centrin4 on the bi-lobed structure in *Trypanosoma brucei*. *Mol. Microbiol*., **2012**, *6*, 1153-61.

[SR36] Wingard, J.N.; Ladner, J.; Vanarotti, M.; Fisher, A.J.; Buchanan, K.T.; Engman, D.M.; Ames, J.B. Structural insights into membrane targeting by the flagellar calcium-binding protein (FCaBP), a myristoylated and palmitoylated calcium sensor in Trypanosoma cruzi *J. Biol. Chem*., **2008**, *283*, 23388-23396.

[SR37] Zhou, J.; Yang, F.; Leu, N.A.; Wang, P.J. MNS1 Is Essential for Spermiogenesis and Motile Ciliary Functions in Mice. *PLoS Genet*., **2012**, *3*, e1002516.

[SR38] Li, J.; Sun, Z. Qilin is essential for cilia assembly and normal kidney development in zebrafish. *Plos One*, **2011**, 6(11):e27365.
